# Immunophenotypic characterisation of non-Hodgkin lymphomas at a tertiary hospital in Ghana

**DOI:** 10.3332/ecancer.2022.1458

**Published:** 2022-10-31

**Authors:** Joseph Adomako, Afua O D Abrahams, Yvonne A Dei-Adomakoh

**Affiliations:** 1 G2 Medical Laboratory Services, 37, Accra, GA-007-6041, Ghana; 2 Department of Pathology, University of Ghana Medical School, University of Ghana, Accra, GA-221-9384, Ghana; 3 Department of Haematology, University of Ghana Medical School, University of Ghana, Accra, GA-221-9384, Ghana

**Keywords:** non-Hodgkin lymphomas, immunohistochemistry, subtypes, diffuse large B cell lymphoma, Ghana

## Abstract

**Background:**

Non-Hodgkin lymphomas (NHLs) are a heterogeneous group of clonal lymphoid tumours originating from lymphocytes. They constitute about 90% of an estimated 3%–4% worldwide distribution of malignant lymphomas among various cancers. Despite the continuous rise and associated deaths, research on NHLs, and in particular the area of immunophenotypic spectrum is limited in Ghana and sub-Saharan Africa.

**Methods:**

A retrospective, descriptive study in which archived tissue blocks of histologically diagnosed NHLs at a tertiary hospital in Accra, Ghana, were used. Antigenic phenotypes were determined by immunohistochemistry.

**Results:**

A total of 66 cases of NHLs, with a mean age of 50.2 ± 16.1 years, were selected for the study. Among the targeted markers, cluster of differentiation 20 (CD20) was the most commonly expressed in 89.4% (59) cases. Immunohistochemistry studies revealed a greater proportion of B cell lymphomas of 89.4%. Five subtypes were successfully identified, of which diffuse large B cell lymphoma constitutes the predominant group (40.9%). A significant association was observed between phenotypic cell types and outcomes of NHLs (*p* = 0.011).

**Conclusion:**

Adult NHLs were mostly due to the malignant transformation of B cells with diffuse large B cell lymphoma being the commonest subtype. The present study therefore serves as preliminary data for further research towards the adoption of an improved treatment regimen and management of NHLs.

## Introduction

Non-Hodgkin lymphomas (NHLs) are a heterogeneous group of clonal lymphoid tumours origi­nating from B cell, T cell or natural killer (NK) lymphocytes. NHLs significantly increase with age, with a peak incidence age of 55–60 years [8].

The global cancer statistics by the Global Cancer Observatory (GLOBOCAN) estimate 544,000 new cases of NHLs and 260,000 deaths due to NHLs in 2020 [16]. Haematological malignancies represent about 7% of all cancers worldwide [13]. Malignant lymphomas which comprise NHLs and Hodgkin Lymphomas (HLs) make up an estimated 3%–4% worldwide of the distribution of malignancies [9]. NHLs make up 80%–90%, and HLs account for the remaining of all malignant lymphomas [5]. They are the most common haematological malignancy and are currently the fifth most common cancer diagnosis among patients in some developed countries [8]. A national strategy for control of cancer document developed by the Ministry of Health, Ghana, showed that out of 830 adult haematological malignancies recorded over a decade at the Haematology/Oncology clinic, Korle Bu Teaching Hospital (KBTH), a tertiary hospital in Accra Ghana, NHLs recorded the highest frequency of 218 (26.3%) [12]. A retrospective review of already diagnosed NHLs of patients aged 13 and above at the same unit, in a period of 4 years and 10 months showed a dramatic figure of 279 [6].

The study sought to determine the immunophenotypic patterns of NHLs by immunohistochemistry and to characterise the NHLs according to pathological cell and subtypes. Association between cell types and outcomes of NHLs was also determined.

Diversity of NHLs requires the determination of a particular cell type and disease sub-type in order to maximise treatment through the selection of the most appropriate therapy. However, diagnosis and treatment are largely based on morphology due to limited resources and financial constraints. Dei-Adomakoh *et al* [6] reported 17 out of 279 (only 6.1%) NHL cases with phenotypic studies. Research on the NHLs and the immunophenotypic distribution is limited in Africa. As of now, not much systematic scientific assessment has been carried out on NHLs in the area of immunophenotypic spectrum in Ghana. There is therefore no clear-cut data on the distribution of phenotypic cells and sub-types necessary for policy directions on local treatment protocols.

## Materials and methods

This was a retrospective, descriptive study in which archived formalin-fixed paraffin-embedded tissue blocks of morphologically diagnosed NHL cases were used. The cases of patients aged 15 and above were received at the Haematology Department, KBTH, Accra, Ghana, between 2015 and 2019.

A data abstraction form was used to retrieve personal, laboratory and clinical information from the folders of patients aged 15 and above. Archived tissue blocks and haematoxylin and eosin (H&E) stained slides of these patients were retrieved at the Pathology Department, KBTH.

Selected tissue blocks were sectioned at 4 microns and mounted on positively charged slides.

Immunohistochemistry studies were performed using BenchMark GX automated immunohistochemistry/in situ hybridization (IHC/ISH) staining instrument (Tucson, VENTANA-Roche, USA). Monoclonal antibodies, **anti-CD20** (L26, VENTANA-Roche, Tucson, USA), **anti-CD3** (2GV6, VENTANA-Roche, Tucson, USA), **anti-CD5** (SP19, VENTANA-Roche, Tucson, USA) and **anti-CD23** (SP23, VENTANA-Roche, Tucson, USA) were used with **OptiView DAB IHC detection kit** [2].

### Examination of slides

Immunohistochemical stained slides were examined under transmitted light illumination. Two pathologists scored the slides independently based on the colour intensity and the percentage of involved tumour cells. Final scores were then determined by averaging independent scores. Discordant scores were re-examined by a third, independent individual and final score made from the two closest.

### Data analysis

Data were entered into 2016 Microsoft excel and analysed using Statistical Package for Social Science version 22. A summary was presented using the descriptive statistics of mean, median, standard deviation and frequency of variables. Graphical displays such as pie chart, frequency distributions or scattergrams, were created where appropriate. Pearson’s chi-square test was used to determine associations between phenotypic cell types (based on immunological markers) and treatment outcome. In cases of sparse data, the Fisher’s exact test was used. All tests were two-sided and a *p*-value less than 0.05 was interpreted as significant.

## Results

A total of 66 cases of NHL were successfully selected for immunohistochemistry studies. The youngest age recorded for the study was 16 years while the oldest was 78 years. The average age of participants in this study was 50.2 ± 16.1 years. Majority 45.5% (*n* = 30) of the participants fall within 41–60 year group. More than half 60.6% (*n* = 40) of the participants were males. These are shown in Table 1.

### Antigen expression in various NHLs

Markers used in the study were CD3, CD5, CD20 and CD23. CD3 antigens were expressed in only 10.6% (*n* = 7) of cases. Also, 31.8% (*n* = 21) were positive for CD5 antigens. Majority 89.4% (*n* = 59) of the cases were positive for CD20. Additionally, 28.8% (*n* = 19) of the cases were positive for CD23 antibodies. These are shown in Table 2.

### Characterisation of NHLs according to cell type

**Figure 1.**
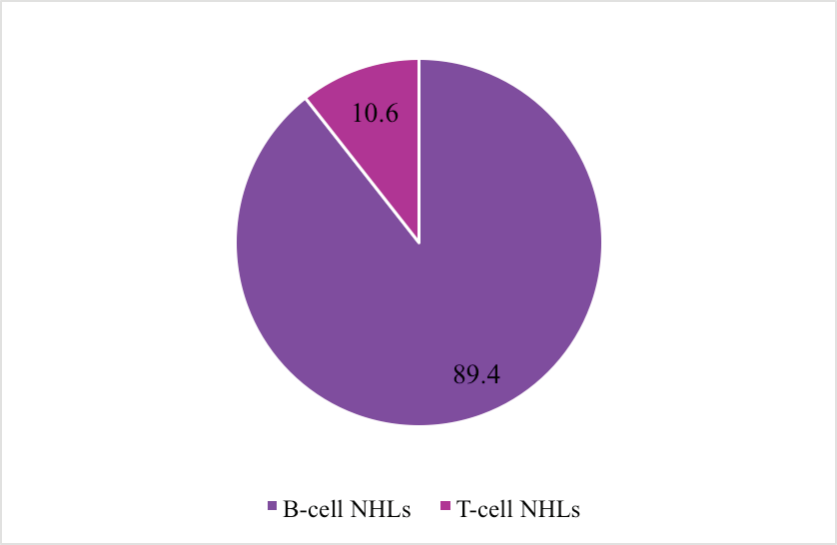
Distribution of NHLs by cell type.

Figure 1 shows the distribution of NHLs according to pathological cell type. Majority 89.4% (*n* = 59) were due to malignant transformation of B cells.

### Phenotypic distribution of NHLs

The commonest subtype 40.9% (*n* = 27) observed in the study was diffuse large B cell lymphoma. Small lymphocytic lymphoma was identified in 12.1% (*n* = 8) cases. Also, significant proportion 7.6% (*n* = 5) of cases were mantle cell lymphoma. T cell lymphomas were mostly diffuse large forms with 7.6% (*n* = 5). However, substantial percentage of B cell lymphomas 25.8% (*n* = 17) were not classified into specific subtypes. These are shown in Table 3.

### Histopathological distribution of NHLs

Table 4 shows that diffuse large forms were the majority of the histopathologically diagnosed NHLs constituting more than 33.3% (*n* = 22), and followed by small cell NHLs, 18.2% (*n* = 12).

### Selected images of slides of NHLs

**Figure 2.**
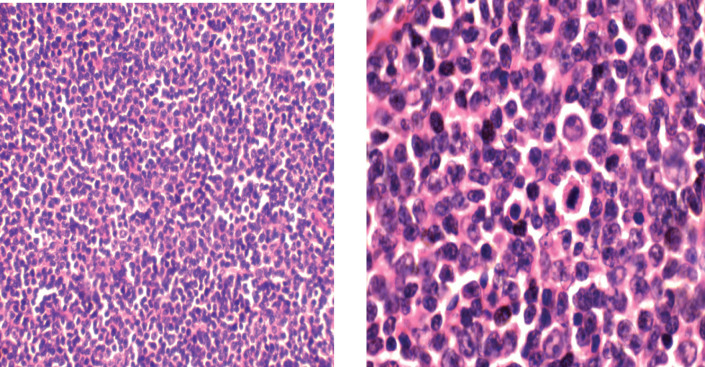
Photomicrograph of diffuse large cell lymphoma.

In Figure 2, H&E (×10, Left; ×40, right) showing a diffuse proliferation of large lymphoid cells. The cytoplasm is moderate to abundant, nuclei are irregular and nucleoli conspicuous. Mitotic figures are frequent.

**Figure 3.**
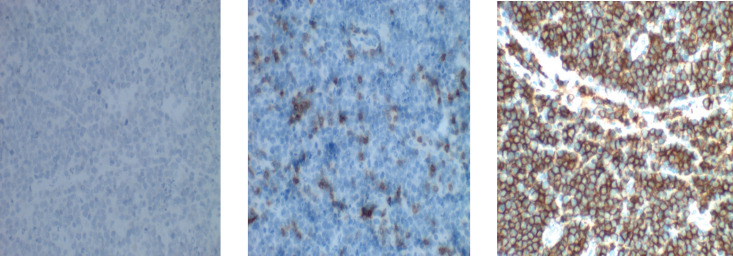
Photomicrograph of B-cell lymphoma.

Immuno-histochemical staining in Figure 3 shows strong membranous positivity for CD20. (×40, right). CD 3 staining (×10, left) is negative and CD 5 staining (×40, middle) highlights the reactive lymphocytes present.

**Figure 4.**
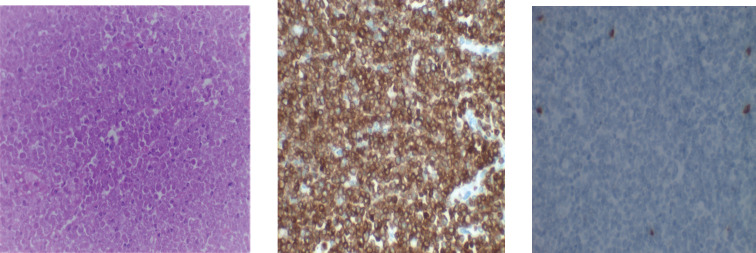
Photomicrograph of T-cell lymphoma.

In Figure 4, H&E (×40, Left) showing a diffuse proliferation of variably sized lymphocytes with irregular nuclei; the neoplastic cells show strong cell membrane positivity to CD 3 (×40, middle) and do not express CD 20 (×40, right)

### Cell types and outcomes

In this study, 37.3% (*n* = 22) of those with B cell lymphomas had clinical remission. A remarkable figure of 15 out of the 22 had completed at least a sixth treatment cycle for the cyclophosphamide, hydroxydaunorubicin hydrochloride, vincristine (oncovin) and prednisone (CHOP) regimen. A proportion of 14.7% (*n* = 1) of those with T cell lymphomas had clinical remission. Also, only 1.7% (*n* = 1) of those with B cell lymphoma had a relapse while 28.6% (*n* = 2) of those with T cell had a relapse. There was a significant association between cell types and clinical outcomes (*p* = 0.011). These are shown in Table 5.

## Discussion

Immunohistochemistry studies throw more light and confirm already morphologically diagnosed NHLs. This study was able to determine the phenotype of abnormal forms revealed by morphology and further characterised these neoplastic populations according to specific lymphoid lineage.

Age and sex are known predisposing factors associated with NHLs according to Alyahya *et al* [3]. For the purpose of this study, the age of participants were put into adolescent and young adults (AYAs) (15–40) and older adults groups based on risk and guidance from National Cancer Institute, USA. Older adults were further put into two groups (41–60 and >60). The mean age was 50.2 ± 16.1 years and comparable to a previous report done in the same unit by Dei-Adomakoh *et al* [6]. Again, age distribution shows that a greater proportion (45.5%) of participants were within 41–60 older adult year group. Together, these indicate a relatively early onset of disease contrary to reports from Saudi Arabia and United states by Alyahya *et al* [3] and Teras *et al* [17], respectively, which follow the pattern that majority of NHLs occur after 60 years. The early occurrence of NHL may be attributed to younger age distribution among the population and personal risk factors. Majority of Ghana’s population is between 15 and 59 years according to the Ghana Statistical Service [11].

Meanwhile, the study observed majority (72.8%) of cases in the older adult group as a whole and therefore suggests that NHL generally increases with age. Additionally, this agrees with the data generated by the National Cancer Institute’s Surveillance, Epidemiology, and End Results Program (SEER), USA, which shows a higher age-standardised rate of 10.5–116.4 per 100,000 in the older adult groups as compared to 1.8–7.2 per 100,000 for AYA group [15].

In the study, males outnumbered females with 1.54:1 male to female ratio. This observation is in agreement with other authors (Onwubuya *et al* [14]; Yakubu *et al* [18]; *Alyahya et al* [3]. Also, male predominance was evident in both B and T cell types, although B cell lymphomas especially subtypes such as diffuse large B-cell lymphoma (DLBCL) and Burkitts lymphoma occur more in males according to SEER [7].

The greater occurrence of NHLs in males may be attributed to socioeconomic factors such as occupation. Thus, the issue of sex-segregation continues to linger in labour especially in the traditional (informal) occupations in Ghana according to Abukari & Odai [1] such that certain male dominated jobs including pesticide applicators, grain millers, wood and forestry workers and farmers may be associated with higher NHLs in men in the study. Moreover, Horesh and Horowitz [7] linked increasing number of pregnancies and live births as well as sex hormones such as oestrogen to the reduction in the risk of developing NHLs in females.

This work mainly utilised the pattern of expression of markers of differentiation. A panel of differentiation markers CD20, CD23 and CD5 were used to confirm neoplasms of B cells while T cell neoplasms were identified with CD3 and CD5. CD20, a pan-B cell maker, was observed in 59 (89.4%) of tumours while 27 of these co-expressed CD23. There was simultaneous co-expression of CD5 with some CD3 and largely with some CD20 proteins. CD3, a pan T cell marker, was least expressed (10.6%). This is similar to findings in a previous study where at least CD20, CD5, CD23 and CD3 markers were used. CD20 was the commonest expressed marker (81.3%) followed by CD5 (50%). However, CD23 was the least expressed in that study [4].

The immunophenotypic findings show that 89.4% of the NHLs were due to malignant transformation of B cells, whereas relatively few cases (10.6%) were derived from T cells. Research work done by Onwubuya *et al* [14], Perry *et al* [19], Sharma *et al* [20], Jairajpuri *et al* [21] and Laurini *et al* [22] in Nigeria, South Africa, India, Iran and Central and South America, respectively, indicates overall greater proportions of B cell lymphomas and hence, consistent with the findings of this study. Documented B cell lymphomas for these studies ranged from 66% to 98.8%. Despite this similarity, differences are observed in the incidence rates of B and T cell lymphomas. T cell lymphomas reported by Jairajpuri *et al* [21] were about three times higher (34%) than that reported in my study while Onwubuya *et al* [14] had only 1.2%. The disparities may be attributed to different geographical locations with varied but related underlying risk factors. For example, some infectious agents [such as *H. pylori*, Human T-lymphotropic virus type 1 (HTLV-1) and HIV/AIDS] are known to be strongly associated with particular NHLs such that higher prevalence of those infections in various locations may correspond to the development of greater proportion of specific cell and sub-types of NHLs. Karin [10] associates much higher adult T cell lymphoma in Japan and Caribbean to the endemic nature of HTLV-1 in these locations. Karin [10] also reports frequent occurrence of some rare T-cell neoplasms in Asia than other continents.

Moreover, in this study, NHLs were put into broad subtypes based on immunophenotype and knowledge on the relevant clinical features and morphology. CD20 positive diffuse large forms were broadly categorised as diffuse large B cell lymphomas without specific division into germinal center B-cell (GCB) and activated B cell (ABC)-like types. CD20 positive small B cell neoplasms with simultaneous co-expression of CD5 and CD23 were identified as small lymphocytic lymphoma. Mantle cell lymphomas were confirmed by the expression of CD20 and CD5 without CD23. About a quarter of B cell neoplasms (25.8%) with morphology and features other than the ones explained above could not be further categorised beyond the cell types. Also, CD3 positive diffuse large cell types and others identified as T cell lymphomas in the study could not be classified into specific subtypes. The vast majority of subtypes recorded were diffuse large B cell lymphoma (40.9%). Although other subtypes, small lymphocytic lymphoma (12.1%), mantle B cell lymphoma (7.6%), extra-nodal B cell marginal zone lymphoma (1.5%) and lymphoplasmacytic lymphoma (1.5%) were observed in the study, ranking of rate of occurrences cannot be appropriately done as significant proportion (25.8%) remain unclassified. The findings of the study are consistent with previous work in Nigeria by Onwubuya *et al* [14] and in India by Atri *et al* [4]. Onwubuya *et al* [14] reported 47% for DLBCL while Atri *et al* [4] had 66.66% for DLBCL and 3.7% for marginal B cell lymphoma. Conversely, more mantle cell lymphoma (7.4%) than small lymphocytic lymphoma (3.7%) was obtained by Atri *et al* [4] and therefore indicates geographical locations with increased possible triggers of particular lymphoma types.

Treatment outcomes were categorised as clinical remission, refractory and relapse. Remission status for greater proportion of both B cell (57.6%) and T cell (57.1%) lymphomas could not be determined as patients were lost to follow-up before completion of a required treatment cycle. A distribution of 37.3% and 14.3% for B and T cell NHLs, respectively, was recorded for clinical remission. This is consistent with the second year outcome of a 3-year follow-up on the outcomes of NHLs by Dei-Adomakoh *et al* [6], which recorded an impressive outcome (78.9% for clinical remission) at the first year with a sharp decline in clinical remission (31.5% for second year and 11.8% for third year) in the following years due to loss to follow-up. Furthermore, a significant association between cell types and clinical outcome was observed for the study (*p* = 0.011), which indicates a varied treatment response and behaviour of NHLs as a result of heterogeneous cell types.

Additional immunological markers including CD10, B cell lymphoma (BCL) 2, BCL6, multiple myeloma oncogene (MUM) 1, CD79a, CD2, CD30 and TdT of B or T/NK cell lineage would have been useful to determine the distribution according to specific subtypes. Clinical outcome of some selected cases could not be determined due to failure of clinical attendance and treatment discontinuation. Co-morbidities such as HIV which would have had an impact on outcomes were not included in the data analysis as it was not routinely done at our department until 5 years ago. In spite of the limitations, the reliability and validity of the study was not compromised.

## Conclusion

NHLs were mostly due to the malignant transformation of B cell lineage with phenotypic distribution of 89.4% for B cells and 10.6% for T cells. The majority of B and T cell lymphomas occurred in males. The predominant subtype observed in the study was diffuse large B cell lymphoma. A significant association was observed between phenotypic cell types and outcomes of NHLs. The present study therefore serves as preliminary data for further research towards the adoption of an improved treatment regimen and management of NHLs in Ghana.

## Conflicts of interest

The authors declare no competing financial interests.

## Figures and Tables

**Table 1.** Demographic characteristics of participants.

Characteristics	Frequency	Percent
Age group (years)		
≤40	18	27.3
41–60	30	45.5
>60	18	27.3
Total	66	100
Sex		
Male	40	60.6
Female	26	39.4
Total	66	100

**Table 2.** Immunophenotypic pattern of NHLs.

Markers (antigens)	Frequency	Percent
CD3	7	10.6
CD5	21	31.8
CD20	59	89.4
CD23	19	28.8

**Table 3.** Distribution of subtypes of NHLs.

Phenotypic subtype	Frequency	Percent
B cell NHLs		
Diffuse large B cell lymphoma	27	40.9
Small lymphocytic lymphoma	8	12.1
Mantle cell lymphoma	5	7.6
Extranodal-marginal zone lymphoma	1	1.5
Lymphoplasmacytic lymphoma	1	1.5
Other B cell types	17	25.8
T cell NHLs		
Diffuse large T cell lymphoma	5	7.6
Other T cell types	2	3.0
Total	66	100

**Table 4.** Diagnosis of NHLs by morphology.

Morphology diagnosis	Total
Burkitt-like	1 (1.5)
Diffuse large and small cell lymphoma	4 (6.1)
Diffuse large cell lymphoma	22 (33.3)
Diffuse large cell lymphoma with CNS involvement	1 (1.5)
MALT lymphoma	3 (4.6)
NHL-Anaplastic large cell type	1 (1.5)
NHL with CNS involvement	2 (3.0)
NHL, lymphoplasmacytic type	1 (1.5)
NHL	10 (15.2)
NHL (follicular type)	7 (10.6)
NHL (large cell type)	1 (1.5)
NHL (small cell type)	12 (18.2)
Small and large cell lymphoma (follicular type)	1 (1.5)
Total	66 (100)

CNS: central nervous system; MALT: mucosa-associated lymphoid tissue

**Table 5.** Association between cell types of NHLs and clinical outcomes.

Cell types			Clinical outcome				Total	χ^2^	*p*-value
			No remission status	Clinical remission	Relapse	Refractory			
B cell	Treatment	CHOP (<6)	12	0	0	0	12	11.13	0.011^a^
		CHOP (6-8)	9	15	1	0	25		
		R-CHOP (6-8)	0	2	0	0	2		
		Chlorambucil	3	1	0	1	5		
		CVP (<6)	8	0	0	0	8		
		CVP (6–8)	1	3	0	0	4		
		CVP (8) + radiotherapy	0	0	0	1	1		
		EPOCH (6–8)	1	1	0	0	2		
	Total		34 (57.6)	22 (37.3)	1 (1.7)	2 (3.4)	59 (100.0)		
T cell	Treatment	CHOP (<6)	1	0	0	0	1		
		CHOP (6–8)	1	1	1	0	3		
		CVP (<6)	2	0	0	0	2		
		EPOCH (8)	0	0	1	0	1		
	Total		4 (57.1)	1 (14.3)	2 (28.6)	0.0 (0.0)	7 (100.0)		
Total (B & T cells)			38 (57.6)	23 (34.8)	3 (4.6)	2 (3.0)	66 (100.0)		

a Significant at 5%

R-CHOP, Rituximab, cyclophosphamide, hydroxydaunorubicin hydrochloride, vincristine (oncovin) and prednisone; CVP**,** Cyclophosphamide, vincristine sulphate, prednisone; EPOCH, Etoposide phosphate, prednisone, vincristine (oncovin), cyclophosphamide, hydroxydaunorubicin (doxorubicin hydrochloride); <6, Received less than six chemotherapy treatment cycle; 6–8, Received 6–8 chemotherapy treatment cycle; 8, Received 8 chemotherapy treatment cycle
